# Editorial: Global excellence in cardiovascular and smooth muscle pharmacology: Europe

**DOI:** 10.3389/fphar.2023.1292234

**Published:** 2023-09-20

**Authors:** Moza M. Al-Owais

**Affiliations:** School of Biomedical Sciences, Faculty of Biological Sciences, University of Leeds, Leeds, United Kingdom

**Keywords:** cardiovascular, smooth muscle, pharmacology, Europe, global excellence

This special edition of Frontiers in Pharmacology focuses on the key emerging research in cardiovascular and smooth muscle pharmacology, to provide insight into current advances in this broad area. In particular, this Research Topic seeks to draw insight between the cardiovascular field and broader considerations for the treatment of conditions such as cancer, Alzheimer’s disease and diabetes.

Heart disease, including cardiovascular disease (CVD), continues to be one of the leading causes of mortality worldwide, causing over 9 million deaths in 2019, with heart attacks and strokes attributed to more than 80% of deaths. Crucially, approximately 30% of these deaths occur prematurely in people under 70 years of age (WHO, 2021).

Europe is the second most affected region, with estimated deaths from heart disease approaching 4 million people per year (Townsend et al., 2022; BHF, 2023). In the United Kingdom, for example, the NHS estimates that there are currently around 7 million people affected by CVDs, pointing towards greater morbidity and an increase in mortality due to heart disease in the near future (NHS, 2023).

A broad range of drugs, with varying mechanisms of action, are used to treat CVDs. These drugs target different cardiovascular factors, such as blood pressure and heart function, and include anticoagulants, angiotensin-converting enzyme (ACE) inhibitors, angiotensin II receptor blockers (ARBs), beta blockers or beta-adrenergic blocking agents, and ion channel blockers. Cardiovascular research offers a fundamental approach to understanding the pathophysiological and biochemical mechanisms of CVD development and provides a unique opportunity to identify new drug targets, contributing to the development of novel therapeutics for CVDs, to facilitate survival and reduce premature morbidity.

However, CVDs are largely preventable and, in most cases, early diagnosis of those at high risk can help in preventing premature death. Accordingly, by identifying those most at risk of developing CVD, through the assessment of lifestyle factors, such as diet and lack of exercise, and adverse changes associated with declined physiological function, for example, due to aging or hormonal imbalances, effective prevention strategies may be implemented, to avoid or reduce the development of CVD. As with many conditions, prevention is as important, if not more so, than treatment once the disease has developed. However, given the range of risk factors and biochemical mechanisms implicated in the development of CVDs, a diverse and multidisciplinary approach to research is required to progress our understanding of the varied pathophysiological bases of these increasingly important conditions. Moreover, the potential for the development of CVD risk arising from therapeutic approaches to other conditions cannot be overlooked, nor can the involvement of CVDs in other pathological states.

In this edition, Wang et al. focus on the heparanase enzyme, which has been implicated as having a role in angiogenesis, and its potential as a therapeutic target in treating pulmonary hypertension. The prolongation of cardiomyocyte hypertrophy, thought to be a mechanism to compensate for cardiac overload, can lead to impaired cardiac function and ultimately heart failure. Pohjolainen et al. found that activation of Protein Kinase C (PKC), a key signalling molecule involved in cardiac hypertrophy, is modulated through the activation of novel PKC isoforms, and propose that the pro-hypertrophic effect of such activators should be considered in the development of PKC-targeted compounds. Tan et al. explore the link between hyperglycaemia and cardiac arrest and the role that elevated blood glucose may play in worsening outcomes for cardiac arrest patients and the potential therapeutic benefits of Empagliflozin, an antidiabetic drug which reduces hyperglycaemia by the highly selective inhibition of the sodium–glucose co-transporter 2. Their findings from the application of Empagliflozin in a rat model of cardiac arrest demonstrate improved ventricular function and survival time, allied with beneficial metabolic changes, pointing towards a therapeutic benefit for Empagliflozin in treating cardiac arrest patients. Similarly, Gallinat et al. investigate the cardioprotective effects of the Parkinson’s disease-associated protein, DJ-1 in a mouse model of myocardial infarction, and propose these effects may be mediated through G-protein-coupled receptor signalling and modulation of the immune response. Steer et al. seek to increase our understanding of the mechanism of action of flecainide, a treatment for arrhythmias associated with catecholaminergic polymorphic ventricular tachycardia (CPVT).

We hope that this Research Topic proves interesting and stimulating reading and serves to illustrate the breadth and depth of current research across the cardiovascular field. In particular, we hope that the research outlined highlights the need to look beyond a narrow research focus, to seek inspiration and development by drawing together research strands from different fields.
